# Efficacy of argatroban plus alteplase according to time from onset to thrombolysis in acute ischemic stroke: a prespecified *post-hoc* analysis of the ARAIS trial

**DOI:** 10.3389/fneur.2025.1582513

**Published:** 2025-06-25

**Authors:** Yu Cui, Er-Qiang Wang, Yi-Han Wang, Hui-Sheng Chen

**Affiliations:** ^1^Department of Neurology, General Hospital of Northern Theater Command, Shenyang, China; ^2^Department of Neurology, The Fuqing Hospital Affiliated of Fujian Medical University, Fuqing, China

**Keywords:** acute ischemic stroke, argatroban, alteplase, OTT, clinical outcomes

## Abstract

**Background:**

The Argatroban Plus Recombinant Tissue-Type Plasminogen Activator for AIS (ARAIS) trial failed to demonstrate the benefit of argatroban in patients diagnosed with acute ischemic stroke who were treated with intravenous alteplase. A *post-hoc* analysis was conducted to investigate whether the time from onset to thrombolysis (OTT) affected the outcomes.

**Methods:**

The included patients were classified into two subgroups according to OTT. The primary outcome was an excellent functional outcome at 90 days, defined as a modified Rankin Scale score of 0 or 1. The efficacy of argatroban plus alteplase was investigated in each subgroup compared with alteplase alone.

**Results:**

Overall, 696 patients were included: 452 were assigned to the OTT < 180 min subgroup, and 244 to the OTT ≥ 180 min subgroup. The treatment effect of argatroban plus alteplase was similar to that of alteplase alone in the OTT < 180 min subgroup (adjusted RD [95% CI] = 0.5% [−5.6 to 6.6%]) and OTT ≥ 180 min subgroup (adjusted RD [95% CI] = −1.3% [−9.8 to 7.1%]). No significant interaction between the treatment efficacy and OTT was found (*p* = 0.75).

**Interpretation:**

The prespecified secondary analysis indicated that the efficacy of argatroban plus alteplase did not differ according to OTT in terms of achieving 90-day excellent functional outcomes compared with alteplase alone.

**Trial registration:**

ClinicalTrials.gov, Identifier: NCT03740958.

## Introduction

Reperfusion therapies are important treatments for patients with acute ischemic stroke, as recommended by the current guideline (1). It is important to achieve successful vessel recanalization to further improve clinical outcomes following reperfusion therapies in acute ischemic stroke (2). However, the proportion of patients who received intravenous alteplase and achieved successful vessel recanalization is approximately one-third (3, 4). Furthermore, neurological deterioration and poor outcome may result from vessel reocclusion that occurs in some patients showing initial recanalization (5). Thus, it is important to reduce the incidence of neurological deterioration and disability after acute ischemic stroke by improving vessel recanalization.

Argatroban has been used in the clinical treatment of acute ischemic stroke in Asia due to its role in inhibiting thrombin-induced vascular events (6, 7). Previous preliminary clinical trials showed the safety profile and effects of argatroban and alteplase on acute ischemic stroke caused by large artery occlusion (8, 9). The Argatroban Plus Recombinant Tissue-Type Plasminogen Activator for AIS (ARAIS) trial failed to demonstrate that intravenous alteplase followed by argatroban could significantly improve clinical outcomes at 3 months (10). Furthermore, the Multiarm Optimization of Stroke Thrombolysis (MOST) study, which was recently published, demonstrated that adjunctive treatment with intravenous argatroban did not reduce post-stroke disability in patients receiving intravenous thrombolysis within 3 h (11). The neutral results may be attributed to the heterogeneous population, including anterior circulation stroke and posterior circulation stroke. The vascular topography was associated with the prognosis of acute ischemic stroke (12), which has been found to affect the efficacy of argatroban in the ARAIS trial (13). It is well known that a longer time from onset to thrombolysis (OTT) is associated with lower proportional benefits after intravenous thrombolysis (14, 15). This study was designed to explore the efficacy of intravenous alteplase followed by argatroban in patients with acute ischemic stroke, according to different OTT scales.

## Methods

### Study design

The present study was a prespecified *post-hoc* analysis, conducted in accordance with the Strengthening the Reporting of Observational Studies in Epidemiology (STROBE) guidelines. ARAIS, registered with ClinicalTrials.gov (NCT03740958), was a multicenter, randomized controlled trial that enrolled 817 participants to investigate the effect of argatroban combined with alteplase in acute ischemic stroke. The inclusion criteria were as follows: a diagnosis of acute ischemic stroke, National Institute of Health Stroke Scale (NIHSS) scores of 6 or higher at randomization, and inclusion within 4.5 h of stroke onset. The key exclusion criteria were as follows: patients with pre-existing disability before stroke (modified Rankin Scale [mRS] scores of 2 or higher), a history of bleeding events in the last 30 days, and other anticoagulant treatments (16). The Ethics Committees of the General Hospital of Northern Theater Command approved the trial (approval number: k 2018 [45]), and the Declaration of Helsinki was followed during the trial. Participants were asked to sign informed consent. In the ARAIS trial, participants from the full analysis set who did not violate the inclusion criteria or meet any of the exclusion criteria were enrolled in the present study.

### Procedures

Based on a previous study, patients were classified into two OTT subgroups according to the division point: OTT < 180 min and OTT ≥ 180 min (15). According to whether they received argatroban after intravenous thrombolysis, patients were further divided into two treatment groups: argatroban plus alteplase and alteplase alone. Detailed information regarding intravenous alteplase administration and adjustments to the argatroban infusion rates was reported in the ARAIS trial (10). Patients in each treatment group received standard treatments based on current guidelines (1). Baseline characteristics of patients were collected at randomization. Neurological status was measured at randomization, as well as at 48 h and 14 days after randomization, using the NIHSS score. Follow-up was completed at 90 days after randomization.

### Outcomes

An excellent functional outcome at 90 days was the primary outcome. Secondary outcomes included favorable functional outcome at 90 days, the distribution on the 90-day mRS score, the incidence of early neurological improvement (17), the incidence of early neurological deterioration (18), the change in NIHSS score at 14 days or discharge (if earlier) compared with baseline, the incidence of stroke or other vascular events, and the incidence of all-cause death within 90 days. Detailed definitions of early neurological improvement and deterioration were consistent with those of the ARAIS trial (10).

**Table 1:** Baseline characteristics between treatment groups.

Characteristics	OTT <180 min			OTT ≥180 min		
	Argatroban plus alteplase (*N* = 209)	Alteplase alone (*N* = 243)	*p*-value	Argatroban plus alteplase (*N* = 120)	Alteplase alone (*N* = 124)	*p*-value
Age, y	65 (58–71)	62 (55–70)	0.12	67 (58–72)	66 (58–71)	0.45
Sex (F)	71 (34.0)	65 (26.7)	0.09	32 (26.7)	34 (27.4)	0.90
Current smoker	72 (34.4)	89 (36.6)	0.79	48 (40.0)	48 (38.7)	0.99
Current drinker^a^	34/202 (16.8)	45/239 (18.8)	0.50	31/117 (26.5)	18/121 (14.9)	0.12
Comorbidities^b^						
Hypertension	129 (61.7)	135 (55.6)	0.19	65 (54.2)	73 (58.9)	0.46
Diabetes	58 (27.8)	46 (18.9)	0.03	31 (25.8)	27 (21.8)	0.46
Previous stroke^c^	40 (19.1)	46 (18.9)	0.96	30 (25.0)	18 (14.5)	0.04
Previous TIA	2 (1.0)	4 (1.6)	0.52	1 (0.8)	0 (0.0)	0.31
Blood pressure at randomization, mmHg						
Systolic	157(140–170)	150 (136–165)	0.02	158 (140–170)	150 (140–172)	0.40
Diastolic	90 (83–100)	88 (80–97)	0.27	90 (80–99)	88 (80–98)	0.55
FBG at randomization, mmol/L	6.80 (5.80–9.72)	6.61 (5.61–8.84)	0.53	6.44 (5.49–9.30)	7.00 (5.73–9.07)	0.29
NIHSS score at randomization^d^	9 (7–12)	8 (6–12)	0.07	9 (7–12)	9 (6–12)	0.49
Estimated premorbid function (mRS score)^e^						
No symptoms (score, 0)	171 (81.8)	199 (81.9)	0.98	92 (76.7)	96 (77.4)	0.89
Symptoms without any disability (score, 1)	38 (18.2)	44 (18.1)		28 (23.3)	28 (22.6)	
OTT, min	128 (100–153)	126 (95–154)	0.64	211 (195–244)	219 (194–240)	0.31
Duration of hospitalization, d	10 (8–13)	9 (7–13)	0.66	10 (7–12)	10 (7–14)	0.35
Presumed stroke cause^f^						
Undetermined	137/206 (66.5)	169/242 (69.8)	0.60	76/119 (63.9)	86 (69.4)	0.27
Large artery atherosclerosis	40/206 (19.4)	43/242 (17.8)		24/119 (20.2)	26 (21.0)	
Small artery occlusion	20/206 (9.7)	16/242 (6.6)		10/119 (8.4)	10 (8.1)	
Cardioembolic	9/206 (4.4)	13/242 (5.4)		8/119 (6.7)	2 (1.6)	
Other	0/206 (0.0)	1/242 (0.4)		1/119 (0.8)	0 (0.0)	
Vascular topography^g^						
Anterior circulation stroke	89/112 (79.5)	96/126 (7602)	0.77	52/66 (78.8)	46/61 (75.4)	0.52
Posterior circulation stroke	19/112 (17.0)	26/126 (20.6)		13/66 (19.7)	15/61 (24.6)	
Anterior and posterior circulation stroke	4/112 (3.6)	4/126 (3.2)		1/66 (1.5)	0/61 (0.0)	

Data are presented as median (interquartile range) or number (percentage). FBG, fasting blood glucose; mRS, modified Rankin Scale; NIHSS, National Institutes of Health Stroke Scale; OTT, time from symptom onset to intravenous thrombolysis; TIA, transient ischemic attack.

a Defined as alcohol consumption at least once a week within 1 year prior to disease onset.

b The comorbidities were based on the patient or family report.

c Previous strokes included ischemic and hemorrhagic. Previous ischemic stroke was referred only to patients with a pre-stroke mRS ≤ 1.

d Patients with NIHSS scores of ≥6 were eligible for this study; NIHSS scores range from 0 to 42, with higher scores indicating more severe neurological deficit.

e Scores on the modified Rankin Scale (mRS) for functional disability ranged from 0 (no symptoms) to 6 (death).

f Presumed stroke cause was classified according to the Trial of ORG10172 in Acute Stroke Treatment (TOAST) using clinical findings, brain imaging, and laboratory test results. Other causes include nonatherosclerotic vasculopathies, hypercoagulable states, and hematologic disorders.

g Definite conclusions based on vessel examination. The diagnosis was based on the clinician’s interpretation of the clinical features and examination results at the time of hospital discharge.

Any bleeding on the brain CT scan, followed by clinically significant neurological deterioration, was used to define sICH (19). Confluent bleeding occupying over one-third of the infarct volume, followed by mass effect, was used to define PH-2 (20). A drop in the hemoglobin level of 2 g/dL or more, or a blood transfusion of 2 U or more, was used to define major systemic bleeding (10). The three types of bleeding events mentioned above were designated as prespecified safety outcomes.

One investigator, unblinded to the treatment assignment, evaluated the NIHSS scores and defined the bleeding events. Trained investigators from participating centers, blinded to the treatment allocation, evaluated the score and events at 90 days by outpatient or telephone interviews.

### Statistical analysis

The present study included a subset of participants from the ARAIS trial. To detect potential bias, baseline characteristics of patients were compared with the present study and the full analysis set of the ARAIS trial. Furthermore, the present study primarily used adjusted analyses due to unbalanced characteristics between treatments after classification regarding OTT.

With respect to continuous variables and categorical variables, baseline characteristics were summarized as medians (interquartile ranges) and frequencies (percentages), respectively. Excellent functional outcome, favorable functional outcome, incidence of early neurological improvement, incidence of early neurological deterioration, incidence of all-cause mortality, and safety outcomes were described as the absolute number of events (percentages), with treatment effects estimated as the risk difference (RD). Other treatment effects were estimated as odds ratios (OR) for the shift distribution of the mRS score, geometric mean ratios (GMR) for the change in NIHSS score, and hazard ratios (HR) for stroke or other vascular events. All treatment effects were shown with 95% confidence intervals (CIs).

We explored the relationship between the probability of the primary outcome and OTT. The probability was calculated between treatment groups using a binary logistic regression model, and the best-fit line with a 95% CI was drawn. With respect to the continuous characteristics of OTT, we assessed the effect of OTT on the primary outcome by including OTT as a covariate in the analyzed model. In the treatment effect analyses, the models were selected based on the distribution of outcomes. (1) For the binomial distribution of excellent functional outcomes, favorable functional outcomes, early neurological improvement, early neurological deterioration, all-cause mortality, and safety outcomes, as well as the continuous distribution of change in the NIHSS score, generalized linear models were used. (2) For the ordinal distribution of mRS scores, ordinal logistic analysis was conducted. (3) For the time-dependent characteristics of stroke or other vascular events, a Cox regression model was used. We hypothesized that treatment effects were similar between treatment groups and rejected the null hypotheses if the *p*-values were less than 0.05, which represented that treatment effects were significantly different.

In the adjusted analyses, characteristics that showed differences between treatment groups were included in the analyzed models, which used outcomes as the dependent variable, treatment groups as the independent variable, and imbalanced baseline characteristics with a *p*-value of less than 0.1 as covariates. The imbalanced bias from covariates was adjusted to detect the association between treatments and outcomes in each subgroup and to enhance the reliability of the results. No missing data were observed in the covariates included in the adjusted analyses. Interactions between the efficacy of treatments and the OTT were assessed using the models mentioned above, and *P_int_*-values were calculated for interaction effects.

The IBM SPSS software (Version 26.0, SPSS Inc.) was used in the present study.

## Results

A total of 64 participants who violated the criteria of the ARAIS trial were excluded, and 696 participants were included, comprising 452 in the OTT < 180 min subgroup and 244 in the OTT ≥ 180 min subgroup (Supplementary Figure S1). Baseline characteristics of participants were similar between the present study and the full analysis set of the ARAIS trial. Participants in the OTT ≥ 180 min subgroup were older than those in the OTT < 180 min subgroup (median age, 66 vs. 63). Detailed information is shown in Supplementary Tables S1, S2.

In the OTT < 180 min subgroup, 209 participants were assigned to the argatroban plus alteplase treatment, while 243 participants were assigned to the alteplase alone treatment. There were some imbalances in sex (34.0% vs. 26.7%), history of diabetes mellitus (27.8% vs. 18.9%), median systolic blood pressure at admission (157 mmHg vs. 150 mmHg), and median NIHSS score at randomization (9 vs. 8) between the two treatment groups. In the OTT ≥ 180 min subgroup, there were 120 participants assigned to the argatroban plus alteplase treatment and 124 participants assigned to the alteplase alone treatment. A higher percentage of previous strokes was found in participants treated with argatroban plus alteplase (25.0% vs. 14.5%). Detailed information is shown in Table 1.

**Figure 1:**
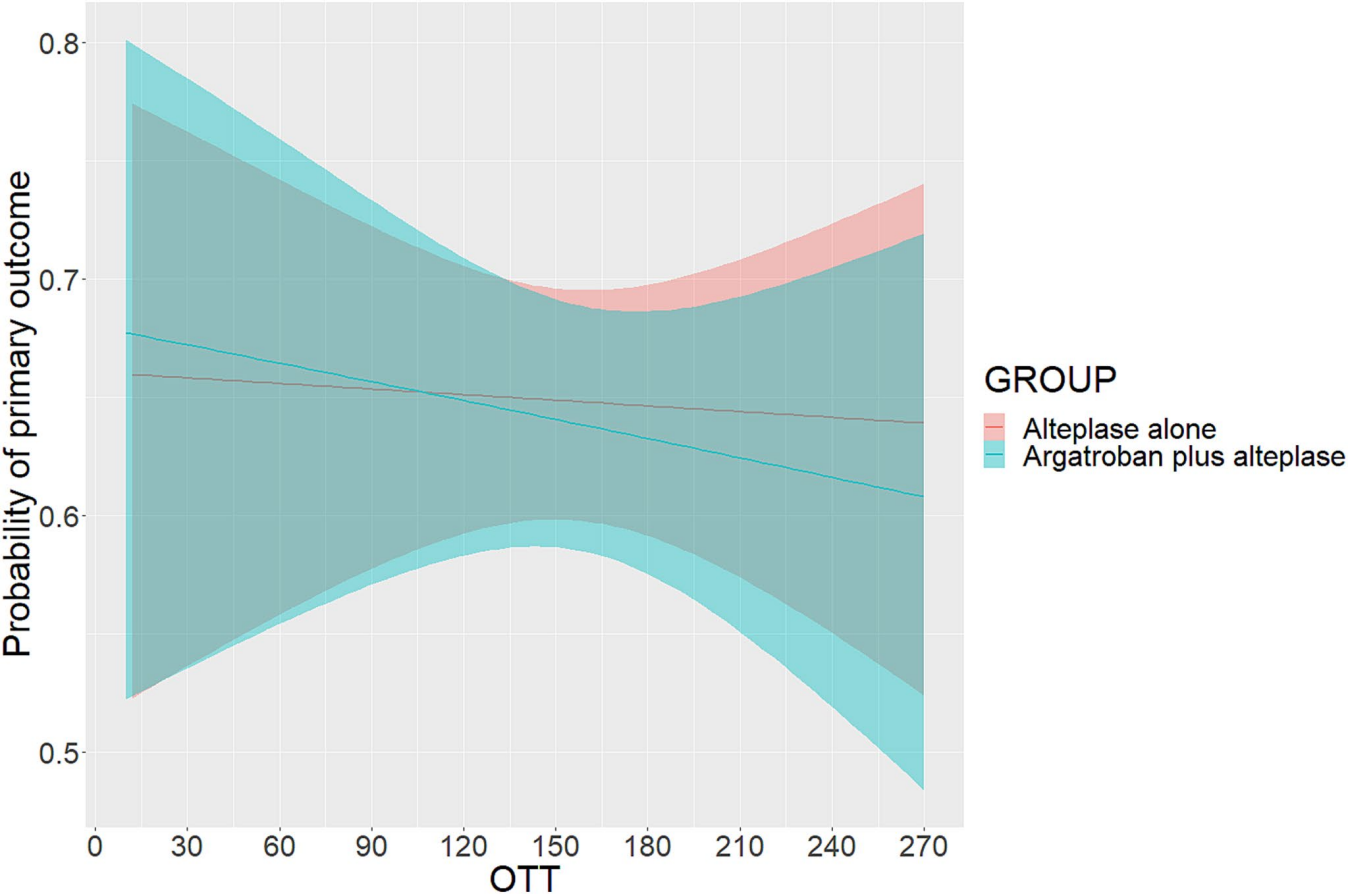
Probability of primary outcome according to OTT. OTT was defined as the time from onset to intravenous thrombolysis. The primary outcome was excellent functional outcome, which was defined as a modified Rankin scale score of 0 or 1 at 90 days.

**Figure 2:**
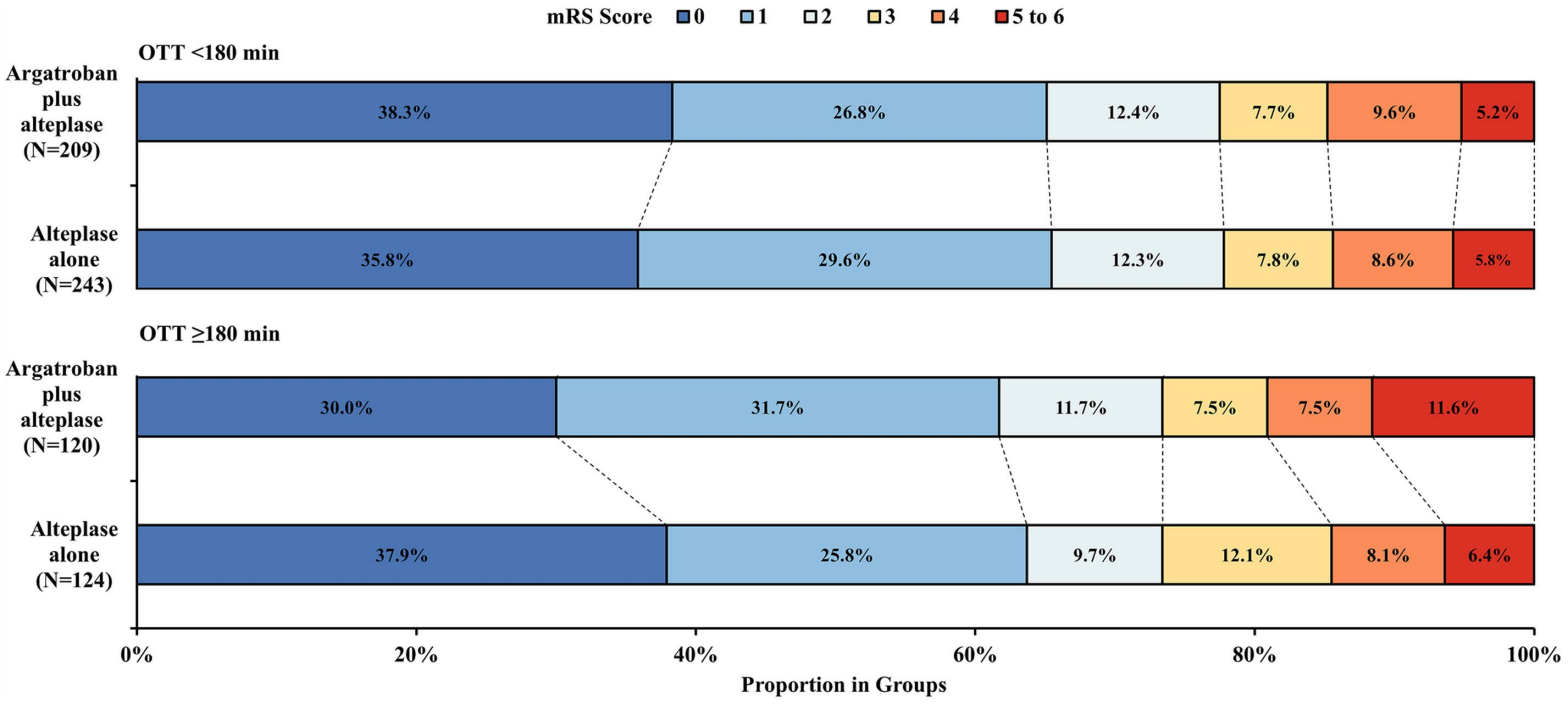
Distribution of mRS score at 90 days. mRS, modified Rankin Scale; OTT, time from onset to intravenous thrombolysis. The mRS scores ranged from 0 to 6. 0, no symptoms, 1 = symptoms without clinically significant disability, 2 = slight disability, 3 = moderate disability, 4 = moderately severe disability, 5 = severe disability; and 6, death.

The probability of the primary outcome decreased as the OTT increased in both treatment groups (Figure 1). We detected the treatment effect on the primary outcome using continuous OTT and dichotomized OTT subgroups. The proportion of participants with the primary outcome in the argatroban plus alteplase and alteplase alone treatment groups was 65.1% vs. 65.4% in the OTT < 180 min subgroup and 61.7% vs. 63.7% in the OTT ≥ 180 min subgroup (Figure 2). Compared with alteplase alone, argatroban plus alteplase had similar odds of achieving the primary outcome across three groups: the overall OTT (adjusted RD [95% CI], −0.9% [−8.1 to 6.2%], *p* = 0.80), the OTT < 180 min subgroup (adjusted RD [95% CI], 0.5% [−5.6 to 6.6%], *p* = 0.88), and the OTT ≥ 180 min subgroup (adjusted RD [95% CI], −1.3% [−9.8 to 7.1%], *p* = 0.75). Furthermore, for the primary outcome, we found no significant interaction effects between treatment groups and OTT subgroups (*P _int_* = 0.75) or OTT (*P _int_* = 0.38). For secondary and safety outcomes, we found neither significant differences between the treatment groups in subgroups nor interaction effects between the OTT subgroups. Additionally, with respect to the neurological causes of death, there were 13 patients in the OTT < 180 min subgroup and 8 patients in the OTT ≥ 180 min subgroup. A total of 4 patients in the OTT < 180 min subgroup and 4 patients in the OTT ≥ 180 min subgroup were attributed to non-neurological causes. Detailed information on the results is shown in Table 2.

**Table 2:** Outcomes comparison between treatments groups.

Outcomes	OTT subgroups	No. (%) of events or median difference		Unadjusted		Adjusted^a^		*P*_int_-value^i^
		Argatroban plus alteplase	Alteplase alone	Treatment difference (95% CI)	*p*-value	Treatment difference (95% CI)	*p*-value	
mRS score of 0 or 1 within 90 days^b^^,^^c^	–	210/329 (63.8)	238/367 (64.9)	−1.0 (−8.1 to 6.1)	0.78	−0.9 (−8.1 to 6.2)	0.80	0.38
	OTT < 180 min	136/209 (65.1)	159/243 (65.4)	−0.4 (−9.2 to 8.4)	0.94	0.5 (−5.6 to 6.6)	0.88	0.75
	OTT ≥ 180 min	74/120 (61.7)	79/124 (63.7)	−2.0 (−14.2 to 10.1)	0.74	−1.3 (−9.8 to 7.1)	0.75	
mRS score of 0 to 2 within 90 days^b^^,^^c^	OTT < 180 min	162/209 (77.5)	189/243 (77.8)	−0.3 (−8.0 to 7.4)	0.95	1.7 (−3.7 to 7.0)	0.54	0.94
	OTT ≥ 180 min	88/120 (73.3)	91/124 (73.4)	−0.1 (−11.1 to 11.0)	0.99	0.6 (−7.1 to 8.3)	0.87	
mRS score distribution at 90 days	OTT < 180 min	–	–	1.05 (0.75 to 1.46)	0.79	1.09 (0.87 to 1.38)	0.45	0.29
	OTT ≥ 180 min	–	–	0.79 (0.50 to 1.24)	0.30	0.82 (0.60 to 1.12)	0.21	
Early neurological improvement within 48 h^c^^,^^d^	OTT < 180 min	154/209 (73.7)	172/243 (70.8)	2.9 (−5.4 to 11.2)	0.49	4.4 (−1.3 to 10.2)	0.13	0.58
	OTT ≥ 180 min	80/120 (66.7)	84/124 (67.7)	−1.1 (−12.9 to 10.7)	0.86	−2.4 (−10.5 to 5.8)	0.57	
Early neurological deterioration within 48 h^c^^,^^e^	OTT < 180 min	8/209 (3.8)	12/243 (4.9)	−1.1 (−4.9 to 2.7)	0.56	−1.4 (−3.9 to 1.2)	0.30	0.86
	OTT ≥ 180 min	5/120 (4.2)	6/124 (4.8)	−0.7 (−5.9 to 4.5)	0.80	−1.1 (−4.7 to 2.4)	0.54	
Change in NIHSS score at 14 days^f^^,^^g^	OTT < 180 min	−0.37 (−0.70 to −0.12)	−0.38 (−0.85 to −0.15)	0.04 (−0.04 to 0.12)	0.33	0.03 (−0.02 to 0.08)	0.28	0.45
	OTT ≥ 180 min	−0.37 (−0.69 to −0.12)	−0.32 (−0.77 to −0.14)	−0.01 (−0.11 to 0.08)	0.78	−0.02 (−0.09 to 0.04)	0.52	
Stroke or other vascular events within 90 days^h^	OTT < 180 min	0/209 (0.0)	0/243 (0.0)	N/A	N/A	N/A	N/A	N/A
	OTT ≥ 180 min	1/120 (0.8)	1/124 (0.8)	1.05 (0.07 to 16.73)	0.97	0.86 (0.05 to 14.23)	0.92	
All-cause death within 90 days^c^	OTT < 180 min	8/209 (3.8)	9/243 (3.7)	0.1 (−3.4 to 3.6)	0.95	−0.3 (−2.8 to 2.1)	0.78	0.86
	OTT ≥ 180 min	7/120 (5.8)	5/124 (4.0)	1.8 (−3.6 to 7.2)	0.52	0.2 (−4.9 to 5.3)	0.94	
Safety outcomes								
Symptomatic intracranial hemorrhage^c^	OTT < 180 min	6/209 (2.9)	3/243 (1.2)	1.6 (−1.0 to 4.3)	0.23	1.9 (−0.1 to 3.8)	0.06	0.16
	OTT ≥ 180 min	1/120 (0.8)	3/124 (2.4)	−1.6 (−4.7 to 1.6)	0.33	−1.4 (−3.5 to 0.7)	0.19	
Parenchymal hematoma type 2^c^	OTT < 180 min	5/209 (2.4)	4/243 (1.6)	0.7 (−1.9 to 3.4)	0.58	1.0 (−0.8 to 2.9)	0.28	0.19
	OTT ≥ 180 min	1/120 (0.8)	4/124 (3.2)	−2.4 (−5.9 to 1.1)	0.18	−2.2 (−4.5 to 0.2)	0.07	
Major systemic bleeding^c^	OTT < 180 min	1/209 (0.5)	1/243 (0.4)	0.1 (−1.2 to 1.3)	0.92	−0.2 (−1.1 to 0.8)	0.72	N/A
	OTT ≥ 180 min	0/120 (0.0)	0/124 (0.0)	N/A	N/A	N/A	N/A	

CI, confidence interval; mRS, modified Rankin Scale; N/A, not applicable; NIHSS, National Institute of Health Stroke Scale; OTT, time from symptom onset to thrombolysis.

a Adjusted for covariates compared between argatroban plus alteplase and alteplase alone treatment groups (*p*-value < 0.1 in each subgroup).

b mRS scores: 0, no symptoms, 1, symptoms without clinically significant disability, 2, slight disability, 3, moderate disability, 4, moderately severe disability, 5, severe disability; and 6, death. An excellent functional outcome was defined as an mRS score of 0–1, and a favorable functional outcome was defined as an mRS score of 0–2.

c Calculated using generalized linear model and presented by risk difference.

d Early neurological improvement was defined as a decrease of 2 on the NIHSS score between baseline and 48 h of 2 (14).

e Early neurological deterioration was defined as an increase of 4 on the NIHSS score between baseline and 48 h, but not as a result of cerebral hemorrhage (15).

f NIHSS scores ranged from 0 to 42, with higher scores indicating greater stroke severity. The log (NIHSS+1) was analyzed using a generalized linear model.

g Calculated using generalized linear model and presented by geometric mean ratio.

h Calculated using the Cox regression model and presented by hazard ratio.

i *P*_int_-value indicates the *p*-value for interaction.

## Discussion

The *post-hoc* analysis of the ARAIS trial divided the enrolled participants into two subgroups according to the OTT, aiming to explore the effect of OTT on the efficacy of the combined treatment. The results demonstrated that OTT had no effect on the likelihood of achieving post-stroke excellent functional outcomes at 90 days in participants receiving argatroban plus alteplase compared to those receiving alteplase alone.

Consistent with the findings reported in a previous study (15), the likelihood of an excellent functional outcome at 90 days decreased as the OTT increased and was lower in participants treated within an OTT of ≥180 min compared to those with an OTT of < 180 min. However, compared with alteplase alone, the efficacy of argatroban plus alteplase was similar, regardless of OTT. In the prespecified subgroup of the MOST trial, the efficacy of argatroban alone compared to placebo was also consistent among OTT subgroups (11). A previous study has shown that patients with large vessel occlusion may benefit from earlier intravenous thrombolysis (21), highlighting the importance of the time window for vessel recanalization. On the one hand, a lower dose of argatroban in the ARAIS trial compared with the MOST trial (100 μg/kg bolus followed by 1 μg/kg vs. 3 μg/kg per minute) may not be sufficient to contribute to vessel recanalization, which partially explains the similar results between different OTT subgroups, although argatroban plus alteplase was previously reported to produce more complete recanalization than alteplase alone (8). One the other hand, participants with milder neurological deficits [a median NIHSS score of 9 vs. 13–19.5 in previous studies (8, 9)] were enrolled in the ARAIS trial, which may be attributed to the fact that large vessel occlusion was not mandatory in the inclusion criteria. A severe neurological deficit was associated with a greater probability of large vessel occlusion, which contributed to this stroke (22). Earlier intravenous alteplase plus argatroban may increase the probability of recanalization in cases of large vessel occlusion. Furthermore, reocclusion occurred frequently following earlier recanalization and led to worse outcomes (3). Argatroban initiated in the early stage was worth investigation, given the finding that it plays an important role in improving vessel recanalization and preventing reocclusion. However, the lower dose of argatroban and the proportion of large vessel occlusion may limit its efficacy. Thus, the findings of this *post-hoc* analysis may be suitable for stroke with mild-to-moderate neurological deficit than those with moderate-to-severe deficit. In this context, the efficacy of argatroban plus alteplase in patients with different OTT is worth further exploration, particularly in those with moderate-to-severe stroke, along with a detailed evaluation of the responsible large vessel lesions.

In the OTT ≥ 180 min subgroup, the proportion of patients with mRS scores of 5 to 6 in the argatroban plus alteplase group was numerically higher than that in the alteplase alone group. On the one hand, the proportion of all-cause death in the argatroban plus alteplase group was a little higher than that in the alteplase alone group. On the other hand, more patients with previous stroke were found in the combined group. A previous study found that patients with previous stroke were more likely to have a poor prognosis (23). Thus, we interpreted that the higher proportion of poor functional outcomes may be attributed to a higher number of patients with previous stroke. However, we cannot ignore the potential bias due to the small sample size in the subgroup.

This study was the first to investigate the effect of OTT on the efficacy of argatroban plus alteplase in acute ischemic stroke, as prespecified in the ARAIS trial. The results demonstrated that OTT had no effect on the association of argatroban plus alteplase with clinical outcomes in acute ischemic stroke compared to alteplase alone; in particular, argatroban did not improve functional outcomes in participants receiving intravenous alteplase, regardless of OTT. This finding does not support the initiation of argatroban within 24 h after intravenous alteplase, although it was found to be safe. This finding is in line with current guidelines, which recommend that anticoagulation treatment should be initiated 24 h after intravenous thrombolysis (1).

However, the present study had several limitations. First, the statistical power was affected by the lower proportion of participants in each OTT subgroup. Given that argatroban plus alteplase resulted in a small treatment effect difference in the 90-day excellent functional outcome compared with alteplase alone in the subgroup analysis, which provided only 5% power based on the current sample size and may induce a risk of Type II error, statistical significance with adequate power would require a very large sample size. Second, fewer participants with stroke caused by large-artery atherosclerosis were included. Therefore, the results may not accurately represent the effect of the combined treatment in stroke participants with large vessel occlusion. Furthermore, as the lower proportion of large-artery atherosclerosis in the OTT < 180 min subgroup compared with the OTT ≥ 180 min subgroup (18.5% vs. 20.6%), patients in the earlier treatment subgroup may derive greater benefit from anticoagulant therapy, which limited the generalizability of the findings. Third, the results need to be validated in a non-Chinese cohort. The findings were interpreted carefully due to the exploratory nature of secondary analysis.

In conclusion, the current analysis did not identify any effect of OTT on the 90-day excellent functional outcomes following argatroban plus alteplase treatment compared to alteplase alone in acute mild-to-moderate ischemic stroke. The efficacy of argatroban plus alteplase treatment according to different OTT should be investigated in patients with acute moderate-to-severe ischemic stroke in future.

## Data Availability

The raw data supporting the conclusions of this article will be made available by the authors, without undue reservation.
